# The Rhizobium-Legume Symbiosis: Co-opting Successful Stress Management

**DOI:** 10.3389/fpls.2021.796045

**Published:** 2022-01-03

**Authors:** Justin P. Hawkins, Ivan J. Oresnik

**Affiliations:** Department of Microbiology, University of Manitoba, Winnipeg, MB, Canada

**Keywords:** rhizobium, legume, symbiosis, stress, pH, osmolarity, oxygen, ROS

## Abstract

The interaction of bacteria with plants can result in either a positive, negative, or neutral association. The rhizobium-legume interaction is a well-studied model system of a process that is considered a positive interaction. This process has evolved to require a complex signal exchange between the host and the symbiont. During this process, rhizobia are subject to several stresses, including low pH, oxidative stress, osmotic stress, as well as growth inhibiting plant peptides. A great deal of work has been carried out to characterize the bacterial response to these stresses. Many of the responses to stress are also observed to have key roles in symbiotic signaling. We propose that stress tolerance responses have been co-opted by the plant and bacterial partners to play a role in the complex signal exchange that occurs between rhizobia and legumes to establish functional symbiosis. This review will cover how rhizobia tolerate stresses, and how aspects of these tolerance mechanisms play a role in signal exchange between rhizobia and legumes.

## Introduction

Rhizobia-legume symbiosis is a well-studied interaction which results in the formation of a plant derived organelle for the purposes of symbiotic nitrogen fixation. Establishment of this interaction occurs through a complex signal exchange which is initiated by the secretion of plant derived flavonoids that are then recognized by compatible rhizobia species (Oldroyd and Downie, 2008; Oldroyd et al., 2011). Recognition of flavonoids results in the production of a lipo-chito-oligosaccharide termed Nod factor (NF) which can be perceived by the host legume (Barnett and Fisher, 2006). This triggers calcium spiking in the inner plant cortical cells, resulting in the division of cells which will form the nodule primordia (Ehrhardt et al., 1996; Shaw and Long, 2003). Nod factor recognition also leads to root hair curling which can trap attached rhizobia and form a curled colonized root hair (Fournier et al., 2008). Infection thread formation can be observed after signals, such as exopolysaccharides or lipopolysaccharides, are recognized. This structure penetrates down toward nodule primordial cells where rhizobia become endocytosed into the cells and enclosed in a symbiotic membrane (Jones et al., 2007). Rhizobia then become bacteroids, which may or may not be terminally differentiated, that functionally serve as a plant organelle to reduce atmospheric nitrogen into ammonia which is subsequently utilized by the host legume.

**Figure 1:**
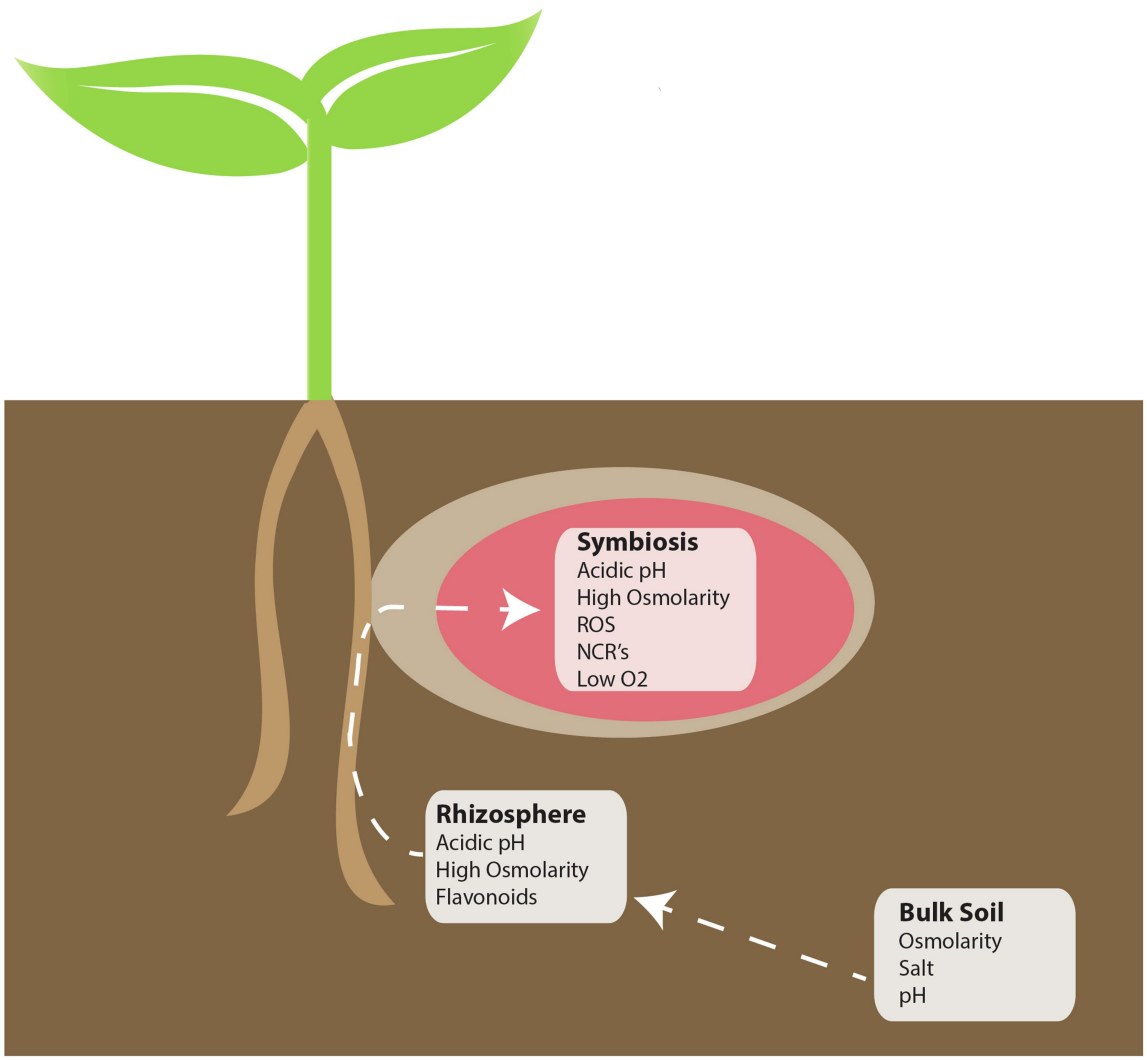
Locations of perceived stress during the rhizobia-legume symbiosis. During symbiosis, there are three distinct environments that symbiotic bacteria must contend with: Bulk soil, the rhizosphere, and *in planta*. Each white box indicates potential “perceived” stressors that may be encountered in each of the indicated environments.

While the signaling events that lead to an effective symbiosis are complex, other factors also play a major role in the establishment of an effective symbiosis. During the infection and differentiation process, rhizobia are challenged by numerous stresses, both in the rhizosphere and *in planta* (Figure 1). To tolerate the stresses that are encountered, bacteria produce compounds or change their lifestyle in order to permit survival. In numerous cases, these changes are correlated with the ability to establish a functional symbiosis. Molecules involved in plant pathogen recognition may also be necessary for symbiotic establishment, and in fact may serve as a signal to the bacteria to produce symbiotic signals. The focus of this work is to review aspects of rhizobia and plant responses to stress, and how elements of these responses may have become co-opted as signals involved in establishing a functional symbiosis.

## Flavonoids

The symbiotic interaction between legumes and rhizobia initiates when flavonoids are recognized by bacteria. The biosynthesis of flavonoids in plants is well understood (Ferrer et al., 2008), and to date, thousands of different flavonoids have been isolated. The biochemical diversity of flavonoids is achieved through modification of a limited number of base structures. These molecules play diverse roles in plant biology ranging from affecting flower color, auxin transport, and anti-microbial defenses (Winkel-Shirley, 2001).

Flavonoids are a known anti-microbial (Hassan and Mathesius, 2012) and represent one of the first directed challenges from plant toward bacteria. The production of these molecules is known to be induced in response to pathogen invasion and has been shown to be directly involved in the plant defense response (Cramer et al., 1985). One subgroup of flavonoids called the iso-flavonoids are found exclusively in legumes (Hirsch et al., 2001). Iso-flavonoids were originally thought to be involved in the defense response against fungi and were shown to also have toxic effects on some isolated bacteria. However, it has also been shown that iso-flavonoids play a role in rhizobium-legume symbiosis. Rhizobia which were exposed to purified iso-flavonoids from plant root exudates were shown to induce the transcription of *nodABC*, which encode proteins necessary for NF synthesis (Hartwig et al., 1990).

Secretion of the flavone luteolin which is produced by *Medicago sativa* has been shown to occur in distinct areas of the developing root where symbiotic interactions may occur (Ehrhardt et al., 1992). Flavones and isoflavones were also found to induce transcription of the *nod* genes in other rhizobia, and recognition of specific flavonoids was shown to play a role in plant-host specificity during symbiosis. Collectively, it seems that the initial role for flavonoids and iso-flavonoids secreted by plants was to be an anti-microbial (Cowan, 1999). However, rhizobia have been able to utilize very specific portions of flavonoids, the iso-flavonoids, as a signal indicating the presence of a compatible host and respond through the production of Nod factor to initiate symbiotic signaling.

## Nod Factor

Signaling between host plants and symbiotic rhizobia or mycorrhizae share a common subset of genes and follow a similar pathway (Duc et al., 1989; Oldroyd, 2013). Each organism produces a lipo-chito-oligosaccharide (*myc*/*nod* factor) that is recognized by LysM type receptors on the plant (Chabaud et al., 2002; Geddes and Oresnik, 2016). Mycorrhizal symbiosis is thought to be an ancient process and able to occur with most land-based plants, with the oldest symbiotic interaction known to be with the phylum Glomeromycota (Oldroyd, 2013). The secretion of *myc* factor, which is structurally similar to nod factor, is essential to its symbiotic interaction with its host plants. Comparatively, rhizobial symbiosis is relatively new and only occurs with legumes and *Parasponia* plants through recognition of NF by LysM type receptors (Pueppke and Broughton, 1999; Madsen et al., 2003). The similarities of the signaling pathway, and insights into *Parasponia* symbiotic signaling, has led to the hypothesis that the use of Nod factor for symbiosis evolved from *myc* factor signaling in mycorrhiza (Streng et al., 2011).

The structure of NF is comprised of 3–5 β(1–4)-linked N-acetylglucosamine residues, with a fatty acid tail on the first residue, and can have various modifications to the N-acetylglucosamine residues (Mylona et al., 1995). Nod factor is structurally similar to fungal cell wall chitin, which is a known activator of the plant immune response (Pusztahelyi, 2018). In addition, both Nod factor and chitin are recognized by LysM type receptors which are thought to have evolved from an ancestral LysM receptor (Zipfel and Oldroyd, 2017). The key difference being that Nod factor contains shorter N-acetylglucosamine chain lengths. In the *S. meliloti* – *M. truncatula* model NF is recognized by the LysM receptors MtNFP and MtLYK3 (Oldroyd, 2013). This recognition induces numerous responses from *M. truncatula* which are necessary for successful symbiotic establishment. Transcriptomic studies have also revealed that Nod factor recognition regulates genes involved in the plant immune response (El-Yahyaoui et al., 2004). Studies have also shown that isolated Nod factor from rhizobia can modulate the immune response of both legumes and non-legumes. When *Arabidopsis thaliana* is exposed to the known pathogen associated molecular pattern *flg22*, the innate immune response of the plant becomes induced. Interestingly, when purified NF isolated from *B. japonicum* was applied, in addition to *flg22*, a more attenuated immune response was observed (Liang et al., 2013). This suggested that Nod factor-mediated suppression of the plants innate immune system may be necessary for successful colonization of plants.

Nod factor is also known to activate the innate defense responses from plants. Transcriptomic studies indicate that the plant immune response is initially activated due to *S. meliloti* inoculum (Lohar et al., 2006). Purified Nod factor has also been shown to induce the production of ROS in the nodulation zone of *M. truncatula* roots (Ramu et al., 2002). Further studies revealed that increased production of H_2_O_2_ can be observed to occur around root hair tips during Nod factor exposure (Cardenas et al., 2008). Interestingly, *S. meliloti* mutant strains over-expressing catalase also exhibited slower nodulation and malformed infection threads (Jamet et al., 2007). This suggests that while the suppression of the plant immune system is necessary for growth of rhizobia during symbiotic establishment, the initial immune response may bring about changes to both plant and bacteria that promote symbiosis. It has been suggested that the presence of H_2_O_2_ might be necessary for stabilizing the formation of infection threads or promoting a physiological change in rhizobia which would promote symbiosis (Jamet et al., 2007). These observations from early signaling involving the interplay of flavonoids and Nod factor production are a clear example of how a potential stress, flavonoids, induce a bacterial signal, Nod factor, that have become a key component for symbiotic interaction.

## Environmental Conditions

The aforementioned topics on symbiotic signaling and regulation of the plant immune response provide a clear example of how tolerance of a stress has become intertwined in signaling. However, it is also important to consider how physiological conditions during symbiotic establishment may also promote symbiosis. Bacteria encounter a wide variety of conditions in both bulk soil and *in planta*. Bulk soil conditions can vary significantly around the world. In addition, the environment *in planta* can challenge bacteria with changes in osmolarity, oxygen content and oxidative stress, decreasing pH, and further plant peptide challenges. The following points investigate how these conditions can promote physiological changes necessary for stress tolerance that end up influencing symbiotic establishment or nitrogen fixation (Table 1).

**Table 1:** Bacterial and plant changes due to perceived stress and their role in symbiosis.

Stress	Response	Symbiotic relevance
Bacteria/Pathogen	Flavonoid	Nod factor induction
	NCRs	Bacteroid differentiation
	Innate immune response	Oxidative burst (see ROS)
Flavonoid	Nod factor	Calcium spiking
Salt/Ion stress	Nod factor	Calcium spiking
	EPS-I	IT development
Osmotic	cyclic β(1–2) glucans	Attachment/ IT formation
	Intracellular Potassium concentration	Nitrogenase induction
Acidic pH	*actR/S*	*fixK/nifA*
	*exoR/S/I*	EPS-I
	Nod factor profile	Legume host range
Reactive oxygen species	EPS-I	IT development
	Membrane crosslinking	IT development
Low oxygen	Intracellular potassium	Nitrogenase induction
	*Fix* genes	Nitrogenase induction
	LPS modification	Legume host range

## Osmotic Stress

Genes involved in adaptation to varying osmotic conditions have been shown to be critical for the establishment of a functional symbiotic relationship. Osmotic conditions before symbiosis are fully dependent on salts and exudates present in the soil. Bulk soil is generally assumed to be an area of low osmolarity (Miller and Wood, 1996). However, the area of the rhizosphere is predicted to have a higher osmolarity due to plant root exudate and water uptake from both plants and bacteria (Jungk, 2002; Miller-Williams et al., 2006). While the osmotic conditions throughout symbiosis in the rhizobia-legume interaction are unknown, current research is consistent with the hypothesis that both high and low osmolarity conditions exist throughout symbiosis (Botsford and Lewis, 1990; Dylan et al., 1990a). The area of the rhizosphere having high osmolarity is particularly interesting as these conditions have been linked with inducing genes necessary for symbiosis. The presence of high osmotic conditions has been shown to induce transcription of genes involved in nodulation and nitrogen fixation (*nod, nif*, and *fix* genes) through NodD2 in *Rhizobium tropici* CIAT 899 (Del Cerro et al., 2019). This same pattern of regulation of these genes through NodD2 is also observed when *R. tropici* is exposed to increased salt stress present in the rhizosphere (Pérez-Montaño et al., 2016). In addition, increased salt concentrations are known to regulate exopolysaccharide production in *S. meiloti* (Miller-Williams et al., 2006). These observations provide the most direct link between osmotic stress recognition and promoting symbiosis.

The major link between osmotic stress tolerance and physiological changes with importance to symbiosis is the accumulation of periplasmic glucans in the presence of hypotonic stress. The majority of the organisms in the family *Rhizobiaceae* produces a cyclic β(1–2)-linked glucan (York et al., 1980; Lelpi et al., 1990; Breedveld and Miller, 1994). Accumulation of these periplasmic glucans can be observed when grown under hypotonic conditions (Miller et al., 1986; Dylan et al., 1990a; Breedveld and Miller, 1995). Further study of cyclic β(1–2) glucans in *S. meliloti* determined that the inability to produce this polysaccharide, by mutating the gene *ndvB*, resulted in sensitivity to hypotonic conditions, and abolished nodule formation on *M. truncatula* (Dylan et al., 1990b). It was hypothesized that extracellular cyclic β(1–2) may be involved in root attachment, but addition of purified cyclic β(1–2) was unable to restore symbiosis with *M. truncatula* to a *ndvB* mutant strain (Dylan et al., 1990b). Pseudorevertants of the *ndvB* mutant strain that still did not produce the cyclic glucan have been isolated and were found to be able to establish a functional symbiosis with *M. truncatula*. However, these suppressors were still heavily impacted in infection thread formation and were sensitive to hypoosmotic stress (Dylan et al., 1990b). This suggested that while cyclic β(1–2) glucan production is important for osmotic stress tolerance and can be linked to infection thread formation, their role in symbiosis extends past stress tolerance. Suppression of the symbiotic phenotype of *ndvB* mutants was later linked to the production the symbiotically important polysaccharide succinoglycan (Nagpal et al., 1992).This lead to the suggestion that production of succinoglycan might provide just enough osmoprotectant in the form of low molecular weight succinoglycan to allow for survival in the absence of the cyclic glucans. In addition, succinoglycan may provide or mask a signal necessary for symbiosis in the absence of cyclic glucans (Abe et al., 1982; Nagpal et al., 1992). Overall, osmolarity is involved in regulating cyclic β(1–2) glucans which have a role in symbiosis that extends past stress tolerance.

Another mechanism rhizobia and other bacteria utilize to tolerate high osmolarity is the accumulation of ions, such as potassium (Yancey et al., 1982; Csonka, 1989; Botsford and Lewis, 1990; Smith et al., 1994; Miller and Wood, 1996). Interestingly, increased potassium levels lead to an increase in nitrogenase activity in *Bradyrhizobium* sp. 32H1 when grown under low oxygen conditions (Gober and Kashket, 1987). As the bacteroid is predicted to be an area of elevated osmotic stress (Miller and Wood, 1996), this provides a link showing that osmotic stress tolerance may be a signal for the regulation of nitrogenase in the bacteroid through the regulation of potassium concentration.

## Low Oxygen Content

During symbiotic establishment, rhizobia encounter areas of low oxygen concentration in the nodule. Control of oxygen concentration is important for symbiosis since oxygen inhibits the activity of nitrogenase (Hunt and Layzell, 1993). Oxygen levels are controlled through a diffusion barrier to create optimal oxygen concentrations for nitrogen fixation (Hunt et al., 1987). Tight regulation of oxygen concentration in bacteroids also leads to a number of signaling and physiological changes in bacteria, which promote symbiosis and nitrogen fixation. It has been well documented that a low oxygen concentration activates the two-component system FixJL, which in turn increases the transcription of the majority of genes involved in nitrogen fixation (David et al., 1988; Virts et al., 1988). Recent work has shown that there are 3 proteins that act oxygen sensors in *Rhizobium leguminosarum*; hFixL, FnrN, and NifA (Dixon and Kahn, 2004; Zamorano-Sánchez and Girard, 2015; Reyes-González et al., 2016). These proteins are tightly temporally controlled, with hFixL inducing expression of FnrN in zones I and II (meristem zone and invasion zone, respectively) of indeterminate nodules. FnrN then induces expression of *fixNOQP* in zone III (nitrogen fixing zone) when oxygen concentration is near anaerobic (Rutten et al., 2021). The induction the genes necessary for production of nitrogenase in near anaerobic conditions is necessary for function of the protein and is also a clear example of how microaerobic stress acts as a signal for symbiosis.

Oxygen concentration has also been shown to regulate lipopolysaccharide (LPS) synthesis and decoration (Kannenberg and Brewin, 1989; Tang and Hollingsworth, 1998). This is thought to have a role in adaptation to the low oxygen environment. Production and modification of LPS are strain specific and are involved in determining host range for symbiosis in some rhizobia (Via et al., 2016). The ability to produce, or properly modify, LPS has been linked to defects in symbiotic establishment (Keating et al., 2002). As LPS content and decoration are dynamic based upon its environment, it is expected that LPS modification would change during symbiosis. Recent work has also shown that flavonoids can induce changes in decoration of LPS and that these changes are necessary for symbiosis (Broughton et al., 2006). It is possible that low oxygen concentration might contribute to bringing about a change in LPS production and decoration which is necessary for both symbiosis and survival in these conditions.

## Reactive Oxygen Species

In addition to low oxygen concentration, rhizobia encounter reactive oxygen species as part of the innate immune response of the plant, and it can be found throughout symbiotic compartments ranging from the IT to mature nodules (Santos et al., 2001). Formation of ROS from the plant immune response has been shown to be beneficial for symbiotic establishment (Puppo et al., 2013). ROS are generated upon Nod factor recognition and are thought to predominantly occur from the activity of NADPH oxidase (Lohar et al., 2007). Rhizobia utilize a number of mechanisms to deal with potential damage from ROS (Boscari et al., 2013). The importance of ROS scavenging during symbiosis is highlighted by the finding that strains which carry mutations in the genes *katB/C*, which encode for catalases, are impaired in forming bacteroids (Jamet et al., 2003). However, a positive role for ROS in symbiosis has also been observed. When catalase is over-expressed in *S. meliloti*, aberrant IT formation and delayed nodule development are observed (Jamet et al., 2007). While it is unknown exactly how ROS may contribute to symbiosis, two main suggestions have been made; either ROS plays a role in IT development, or ROS induces physiological changes in rhizobium that are necessary for symbiosis (Pauly et al., 2006). Recent work has investigated this further and has shown that ROS produced by PvRbohB in *Phaseolus vulgaris* is important for symbiosis. Cultivars of *P. vulgaris* silenced in expression of PvRbohB displayed abortive infection threads when inoculated with *R. tropici* (Fonseca-García et al., 2021). RNAseq data also revealed changes in carbon metabolism and cell cycle control; both of which can be linked with symbiosis (Geddes and Oresnik, 2016; Fonseca-García et al., 2021).

Consistent with the hypothesis that ROS may act as a signal to bacteria for symbiotic establishment, it has been shown *B. japoncium* exposed to oxidative stress produces an increased amount of exopolysaccharides (Donati et al., 2011). The production of exopolysaccharides (EPS) has long been suggested to be involved in the tolerance of various stresses encountered by bacteria. In *S. meliloti* and *Pseudomonas syringae*, mutants unable to produce EPS have been observed to be sensitive to ROS (Király et al., 1997; Lehman and Long, 2013). Furthermore, it was shown that low molecular weight succinoglycan (EPS-I) is the responsible fraction which scavenges H_2_O_2_ from media in *S. meliloti* (Lehman and Long, 2013). Taken together, oxidative stress is seen to promote the production of exopolysaccharides which are necessary for the tolerance of ROS and critical for symbiotic establishment. Since plants are observed to produce H_2_O_2_ in response to symbiotic establishment, this provides a potential example of how *in planta* conditions promote production of a symbiotic signal.

## Ph Stress

The ability to tolerate acidic pH conditions has largely been studied from the perspective of tolerating acidic soils in the environment. The area of the rhizosphere is predicted to be an area of increased acidic stress, as throughout their life cycle, plants can excrete acidic compounds into the surrounding soil, decreasing the pH of the soil by as much as 2 pH units (Faget et al., 2013). This occurs from the secretion of protons to maintain the net charge across the root membrane and from the secretion of organic compounds (Jones et al., 2003). During the symbiotic interaction between rhizobia and legumes, it has been hypothesized that many plant derived compartments have an acidic pH. The bacteroid and peri-bacteroid space have been predicted to be an acidic compartment, reaching a pH of 4.5 (Fedorova et al., 1999; Pierre et al., 2013). Studies have also determined that the curled colonized root hair is an area of localized acidic pH stress (Geddes et al., 2014). These findings are particularly important as *S. meliloti* is known to have poor survival when medium pH decreases below six (Hellweg et al., 2009; Hawkins et al., 2017).

Transcriptomic studies addressing the response of rhizobium to acidic pH and have revealed large networks regulating multiple genes in response to acidic pH (Hellweg et al., 2009; Guerrero-Castro et al., 2018). The response of rhizobia to acidic pH is primarily regulated though two-component systems, *actR/S* and *chvI*/*exoS*/*exoR* (Dilworth et al., 2000; Fenner et al., 2004). These systems ultimately control the regulation of cytoplasmic pH, or the production of and modification of extracellular elements for pH tolerance components (Cunningham and Munns, 1984; Chen et al., 1993). Regulation of potassium efflux proteins is important for pH tolerance. The potassium efflux system in *R. tropici* has been shown to be regulated by glutathione, since mutants in glutathione synthesis were unable to accumulate intracellular potassium (Riccillo et al., 2000). Potassium concentrations have been shown to regulate nitrogenase activity so this accumulation of K^+^ in acidic conditions may act as a symbiotic signal (Gober and Kashket, 1987). In addition, glutathione is involved in tolerating many environmental stressors, including pH and ROS stress, and has been shown to be produced in increased amounts under acidic conditions (Riccillo et al., 2000; Muglia et al., 2007). Mutations in the synthesis pathway for glutathione are known to result in either a fix^−^ or delayed nodulation phenotype (Harrison et al., 2005).

One physiological response of *S. meliloti* to low pH is the production of the symbiotically important exopolysaccharide EPS-I (Hawkins et al., 2017). Acidic pH is known to be present throughout the symbiotic process, being present in the rhizosphere all the way to bacteroids. Mutants which are unable to produce succinoglycan are unable to establish functional symbiosis with alfalfa. Further investigation has revealed that the succinylation of EPS-I is the critical component of the symbiotic interaction (Mendis et al., 2016). Production of EPS-I is also important for tolerance of low pH and contributes to survival in nodules (Hawkins et al., 2017; Maillet et al., 2020). However, symbiotic defects observed in *exo* mutant strains are likely due to a combination of a loss of pH stress tolerance and loss of proper symbiotic signaling. *S. meliloti* strains that lack *exoK* produce a succinylated high molecular weight EPS-I still exhibit high sensitivity to acidic pH, but only display minor symbiotic defects (Maillet et al., 2020). Collectively, these data suggest that EPS-I plays a role in both stress tolerance as well as symbiotic signaling.

It has also been observed that acid tolerant strains of rhizobia produce more exopolysaccharides than acid sensitive strains under non-stress conditions (Cunningham and Munns, 1984). Interestingly, mutations that resulted in an increased production of exopolysaccharide in *R. leguminosarum* and *S. meliloti* did not result in an increased tolerance to acidic media (Howieson et al., 1988; Reeve et al., 1997). These observations suggest that in terms of stress tolerance, the production of exopolysaccharides may serve an on/off function rather than a gradient of tolerance, and that the increased production of exopolysaccharides due to pH stress may have another role.

The response to low pH is largely mediated through the ExoR/ExoS/ChvI (RSI) system, which has been shown to be upregulated due to acidic pH in *S. meliloti* (Hellweg et al., 2009; Draghi et al., 2016). The RSI system is well studied for its ability to regulate the production of EPS-I and flagella (Cheng and Walker, 1998b; Heavner et al., 2015). It is long known that the production of EPS-I is important for symbiotic interaction (González et al., 1996; York and Walker, 1997; Cheng and Walker, 1998a). The protein ExoS acts as a sensor kinase which directly phosphorylates the response regulator ChvI in response to a signal (Cheng and Walker, 1998b; Yao et al., 2004). This system is regulated through direct binding of the repressor ExoR to ExoS in the periplasm (Chen et al., 2008). Homologs of this system in *Agrobacterium tumefaciens* have been shown to be involved in gene regulation due to acidic pH, and it has been suggested that acidity is a key signal in establishing virulence with plants (Li et al., 2002). Further study of the RSI regulon in *A. tumefaciens* has revealed that at acidic pH the repressor ExoR is degraded, resulting in increased EPS-I synthesis (Heckel et al., 2014). A mechanism for degradation of ExoR in *S. meliloti* has also been shown (Lu et al., 2012). Degradation of ExoR could account for the increase in transcription of *exoR* at lower pH. Taken together, this suggests that the acidic conditions found in the curled colonized root hair leads to the production of EPS-I which is necessary for symbiotic signaling and stress tolerance, making pH a key environmental regulator for symbiosis. Overall, these works suggest that low pH induces the production of glutathione and succinoglycan which are both involved in stress tolerance and symbiosis.

## Nodule-Specific Cysteine Rich Peptides

Recently, there has been interest in a subsect of plant produced anti-microbial peptides (AMPs) called nodule-specific cysteine rich (NCR) peptides for their role in symbiotic establishment (Alunni and Gourion, 2016). AMPs are well studied for their anti-microbial activity (Maroti et al., 2011). The mechanism of action of AMPs involves the disruption of bacterial membranes through interaction with the cell surface and ribosome inactivation. In addition to their anti-microbial activity, it has been suggested that certain AMPs play a role in signaling (Schopfer, 1999).

NCRs are structurally and functionally similar to AMPs; they are predicted to be around 100 amino acids long, contain the conserved cysteine residues for disulfide bridge formation, and are predicted to be largely cationic (Mergaert et al., 2003). These peptides have also been shown to have anti-microbial activity against several organisms, including rhizobia (Haag et al., 2011). However, the presence of the protein BacA in *S. meliloti*, a transporter for AMPs, is observed to be involved in tolerating the challenge with NCRs, whereas mutants in *bacA* were observed to be hypersensitive to the anti-microbial activity *in planta* (Haag et al., 2011).

In *M. truncatula*, there are predicted to be upwards of 300 different NCRs produced by around 600 different genes (Mergaert et al., 2003; Zhou et al., 2013). Only legumes of the inverted-repeat lacking clade (IRLC) are observed to produce NCRs (Mergaert et al., 2006). In these legumes, symbiotic bacteria become terminally differentiated into bacteroids in plant nodules and cannot revert to normal functioning bacteria. Non-IRLC legumes, such as *L. japonicus*, do not produce NCRs, and symbiotic bacteria do not become terminally differentiated (Mergaert et al., 2003). This has led to the suggestion that NCRs are directly involved in the terminal differentiation of symbiotic bacteria. However, it is worth noting that examples of bacteroid differentiation outside of the IRLC legumes are starting to be found. Nodules formed in the *Aeschynomene* – *Bradyrhizobium* symbiotic relationship are found to house differentiated bacteroids with a polyploid genome (Czernic et al., 2015). While *Aeschynomene* sp. do not produce NCRs they have been shown to produce NCR-like peptides that likely play a role in differentiation of bacteroids. Silencing the homolog of *dnf1* in *Aeschynomene evenia*, which is necessary for cleavage of NCRs for transport to the symbiosome and is essential for symbiosis, results in deformed bacteroids (Czernic et al., 2015). In addition, the protein BclA was identified in *Bradyrhizobium sp*. as having weak homology to BacA. BclA was shown to be necessary for formation of bacteroids and was observed to be able to transport the peptide NCR247 from *M. truncatula* (Guefrachi et al., 2015). Taken together, there is good indirect evidence that these NCR-like peptides are used for bacteroid differentiation.

The localization of NCRs suggests much about their role in symbiosis. When NCRs are expressed in the nodule, they are targeted to the symbiotic membrane by the plant secretory system and can also be found within the cytoplasm of bacteroids (Van de Velde et al., 2010). In the same study, it was also shown that a mutation in *M. truncatula dnf-1* prevents targeting of NCRs to the bacteroid and prevented bacteroids from terminally differentiating. Also, when NCR035 from *M. truncatula* was expressed in *L. japonicum*, which is deficient in NCR production, it localized to the symbiosome of bacteroids resulting in the production of a single elongated bacteroid indicative of terminal differentiation (Alunni et al., 2007; Van de Velde et al., 2010). This highlighted the importance of NCRs for symbiotic establishment in the IRLC legumes. More recent studies on NCRs have shown that a mutation in the gene *dnf7*, which encodes for a protein involved in the production of NCR169, is unable to perform BNF in *M. truncatula* (Horváth et al., 2015). Nodules in this mutant were impaired in elongation and triggered early senescence. This was fully complemented by overexpression of NCR169. These studies show the necessity of NCRs in regulating bacteroid differentiation and symbiotic nitrogen fixation.

Microarray analysis has also revealed that NCR recognition may play a role in the bacterial stress response, as well as preventing cell division during symbiosis (Penterman et al., 2014). After exposure of *S. meliloti* to NCR247, the expression of genes involved in bacterial stress response and cellular division was found to be altered in transcription. This includes increased transcription of *rpoH1*, which is involved in regulating genes for acid and heat tolerance, and the two-component systems *exoS-chvI* and *feuP-feuQ*, which are responsible for regulating EPS and cyclic β(1–2) glucan production (Reuber et al., 1990; Griffitts et al., 2008). In line with this, NCR247 has been shown to induce transcription of the *exo* genes for EPS-I production, and high molecular weight EPS-I has been shown to aid survival when exposed to NCR247 (Arnold et al., 2017, 2018). Decreased transcription of cell cycle regulators *ctrA* and *gcrA* was also observed (Penterman et al., 2014). These observations led to the conclusion that NCR recognition may be a bacterial signal that allows for adaptation to *in planta* conditions and increase the production of polysaccharides necessary for symbiosis in addition to its role in bacteroid differentiation. This shows that NCRs may have evolved in plants from simply being an AMP produced as a response to bacterial invasion, to also being involved in symbiotic establishment as a signal which induces physiological and morphological changes in the bacteria necessary for nitrogen fixation.

## Discussion

**Figure 2:**
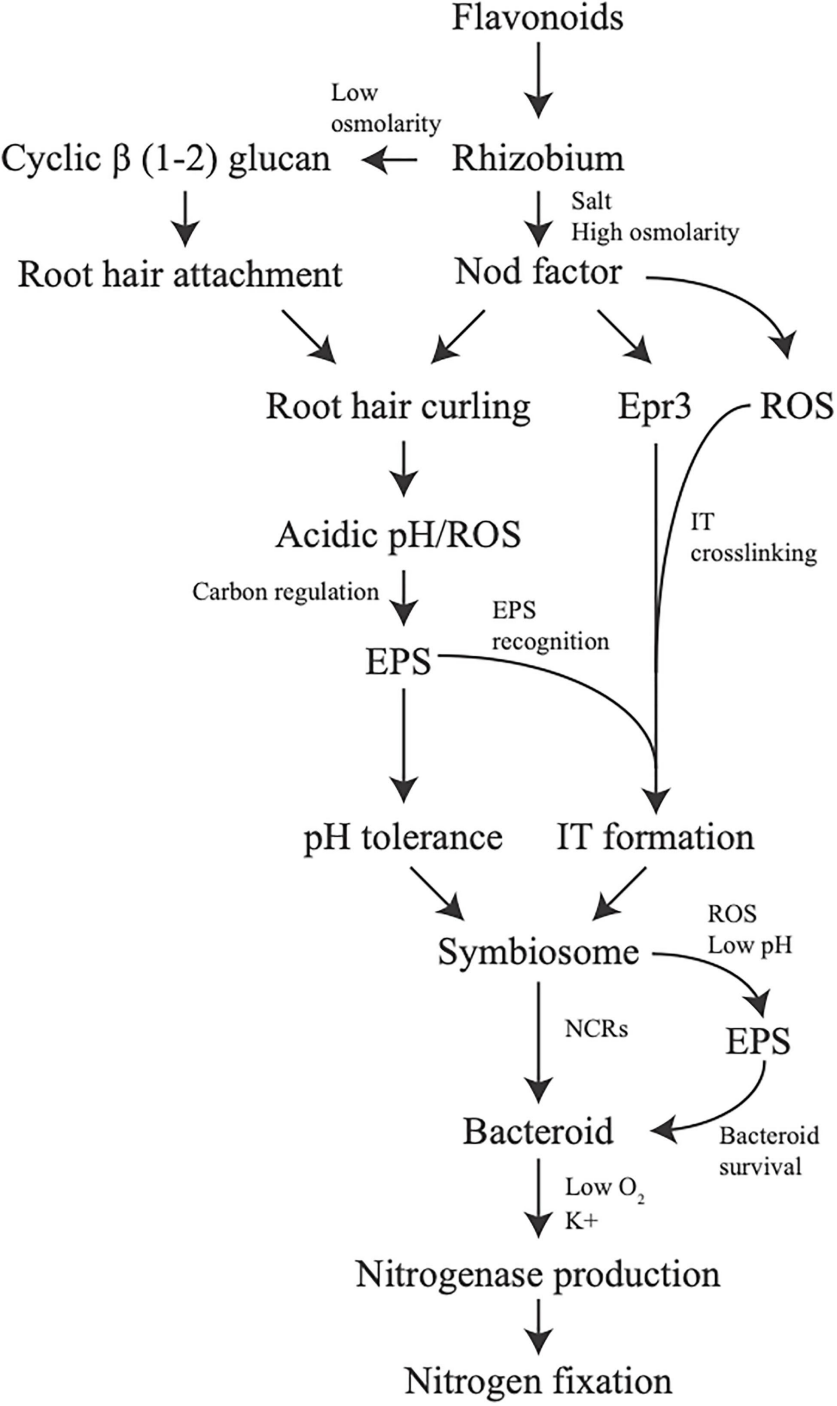
Stress tolerance involvement in symbiotic signaling. Changes in production of molecules or overall physiology due to stress can be observed to affect symbiosis all throughout the process. Each line indicates how a potential stress, or a response from a stress, influences the next step in the symbiotic process.

The establishment of the rhizobium – legume symbiotic interaction is often described as a direct complex signal exchange between both the bacteria and the plant, with emphasis placed on how a molecule from one induces changes in the other or invokes a signaling response. However, little emphasis has been placed on how environmental conditions and stress tolerance play into the interaction. Here, we provide evidence that the tolerance of environmental conditions and challenges by the plant immune system result in alterations of bacterial physiology which promotes establishment of symbiosis between plant and bacteria. This broadens our assumptions of the signaling cross-talk between legume and rhizobia which is largely considered from the perspective of secreted signal and direct response. In addition, we should consider overall physiological changes in bacteria due to conditions in the soil from root exudate or *in planta* as part of the signal exchange symbiosis in addition to the role in surviving the stress conditions. If molecules produced as part of the stress response by bacteria and plant are examined it can be seen that stress plays an important role from the start of symbiosis, all the way to nitrogen fixation (Figure 2).

Some of the examples used are already well studied for their role specifically in symbiosis. This includes iso-flavonoids, Nod factor, and NCRs. While these molecules now have roles directly in symbiotic signaling, their overall origin in this process comes from the immune response of the plant. What likely originated as a stress challenge of anti-microbials for bacteria and fungi with flavonoids and NCRs has turned into critical signals to initiate the symbiotic process or for forming terminally differentiated bacteroids. Cell wall chitin from fungi which is recognized as a PAMP has become inherited by rhizobia in the form of Nod factor, which is now the critical signaling molecule secreted by bacteria to establish symbiosis. The similarities of the responses between either plant immunity or symbiosis are significant and stretch much further past what is discussed here (Berrabah et al., 2015; Tóth and Stacey, 2015; Zipfel and Oldroyd, 2017). It is quite likely that as more detailed mechanisms of each of these responses are uncovered, it will be seen that there is significant cross-talk or similarities linking stress responses and symbiosis. In a number of cases, the difference between either killing the bacteria or establishing a functional symbiosis seems to be based upon the strength of the plant immune response to the organism. A strong response to repel an invader, or an attenuated one to induce physiological changes in a potential symbiont.

Other examples of how potential conditions bacteria may be exposed to in the soil or *in planta* are less directly tied to symbiosis, but the link is still quite clear. A large part of the stress response of symbiotic rhizobia revolves around production or modification of various polysaccharides, such as cyclic β(1–2) glucans, lipopolysaccharides, and succinoglycan. These polysaccharides are also intrinsically linked to symbiotic establishment across a number of different rhizobia-legume interactions. While the role of cyclic β(1–2) glucans in symbiosis is yet unclear, production and proper decoration of LPS and succinoglycan are suggested to be critical signaling molecules to avoid the full activation of the plant immune response (Ojeda et al., 2013; Kawaharada et al., 2015; Maillet et al., 2020). Additionally, there are around 17 different hypothetical operons for polysaccharide production in *S. meliloti* so it is plausible to think other polysaccharides that are yet unclassified may play an important role in the stress tolerance/symbiosis picture as well.

Aside from polysaccharide production, these adverse conditions encountered also changes cell physiology in terms of ion uptake, glutathione production, and shifts in carbon metabolism which can all be linked in some regards to the symbiotic process. It is not hard to imagine that symbiotic bacteria may have evolved its responses over time to stress conditions present in root exudate or *in planta* to start adjusting its physiology for a symbiotic lifecycle. In addition, it is understandable why a plant would evolve to promote certain conditions using root exudate and use an altered immune response if the eventual gain becomes a symbiotic nitrogen fixing bacteria.

One of the major overall goals of nitrogen fixation research is to eventually bring the symbiotic relationship between legumes and rhizobia to non-legume plants, such as the cereal crops. The potential impact this could have in reducing use of nitrogen fertilizers, and for overall growth of plants where fertilizers are not available, is quite significant. While research in this area is new and ongoing, it largely focuses on adjusting and tuning directly observed signaling between rhizobia and these plants. It is important to remember that aside from signaling and adjusting the plant’s immune response to go from immunogenic to symbiotic, the overall environment in the rhizosphere and *in planta* may also play a key role for symbiosis and have to be accounted for. At the end of the day, it is always said that stress, unfortunately, is a great motivator in life. This also seems to be true with respect to the rhizobium-legume symbiosis.

## Author Contributions

JPH and IJO conceived and wrote the manuscript. All authors contributed to the article and approved the submitted version.

## Funding

This work was supported by a Natural Sciences and Engineering Research Council of Canada Discovery grant (RGPIN-2018-04966) to IJO.

## Conflict of Interest

The authors declare that the research was conducted in the absence of any commercial or financial relationships that could be construed as a potential conflict of interest.

## Publisher’s Note

All claims expressed in this article are solely those of the authors and do not necessarily represent those of their affiliated organizations, or those of the publisher, the editors and the reviewers. Any product that may be evaluated in this article, or claim that may be made by its manufacturer, is not guaranteed or endorsed by the publisher.

## References

[ref1] Abe M. Amemura A. Higashi S. (1982). Studies on cyclic β-1,2-glucan obtained from periplasmic space of rhizobium trifolii cells. Plant Soil 64, 315–324. doi: 10.1007/BF02372514

[ref2] Alunni B. Gourion B. (2016). Terminal bacteroid differentiation in the legume - rhizobium symbiosis: nodule-specific cysteine-rich peptides and beyond. New Phytol. 211, 411–417. doi: 10.1111/nph.14025, PMID: 27241115

[ref3] Alunni B. Kevei Z. Redondo-Nieto M. Kondorosi A. Mergaert P. Kondorosi E. (2007). Genomic organization and evolutionary insights on GRP and NCR genes, two large nodule-specific gene families in *Medicago truncatula*. Mol. Plant Micro.Interact. 20, 1138–1148. doi: 10.1094/MPMI-20-9-1138, PMID: 17849716

[ref4] Arnold M. F. F. Penterman J. Shabab M. Chen E. J. Walker C. (2018). Important late-stage symbiotic role of the Sinorhizobium meliloti exopolysaccharide Succinoglycan. J. Bacteriol. 200:e00665-17. doi: 10.1128/JB.00665-17 29632097 PMC5996692

[ref5] Arnold M. F. F. Shabab M. Penterman J. Boehme K. L. Griffitts J. S. Walker C. (2017). Genome-wide sensitivity analysis of the Microsymbiont Sinorhizobium meliloti to symbiotically important, Defensin-Like host peptides. MBio 8:e01060-17. doi: 10.1128/mBio.01060-17 28765224 PMC5539429

[ref6] Barnett M. J. Fisher R. F. (2006). Global gene expression in the rhizobial-legume symbiosis. Symbiosis 42, 1–24. doi: 10.1111/j.1574-6968.1999.tb13650.x

[ref7] Berrabah F. Ratet P. Gourion B. (2015). Multiple steps control immunity during the intracellular accommodation of rhizobia. J. Exp. Bot. 66, 1977–1985. doi: 10.1093/jxb/eru545, PMID: 25682610 PMC4378630

[ref8] Boscari A. Meilhoc E. Castella C. Bruand C. Puppo A. Brouquisse R. (2013). Which role for nitric oxide in symbiotic N_2_-fixing nodules: toxic by-product or useful signaling/metabolic intermediate? Front. Plant Sci. 4:384. doi: 10.3389/fpls.2013.00384, PMID: 24130563 PMC3793596

[ref9] Botsford J. L. Lewis T. A. (1990). Osmoregulation in rhizobium meliloti: production of glutamic acid in response to osmotic stress. Appl. Environ. Microbiol. 56, 488–494. doi: 10.1128/aem.56.2.488-494.1990, PMID: 16348124 PMC183366

[ref10] Breedveld M. W. Miller K. J. (1994). Cyclic β-glucans of members of the family Rhizobiaceae. Microbiol. Rev. 58, 145–161. doi: 10.1128/mr.58.2.145-161.1994, PMID: 8078434 PMC372960

[ref11] Breedveld M. W. Miller K. J. (1995). Synthesis of glycerophosphorylated cyclic (1,2)-β-glucans in rhizobium meliloti strain 1021 after osmotic shock. Microbiology 141, 583–588. doi: 10.1099/13500872-141-3-583, PMID: 7711896

[ref12] Broughton W. J. Hanin M. Relic B. Kopcinska J. Golinowski W. Simsek S.. (2006). Flavonoid-inducible modifications to rhamnan O antigens are necessary for rhizobium sp. strain NGR234-legume symbioses. J. Bacteriol. 188, 3654–3663. doi: 10.1128/JB.188.10.3654-3663.2006, PMID: 16672619 PMC1482867

[ref13] Cardenas L. Martinez A. Sanchez F. Quinto C. (2008). Fast, transient and specific intracellular ROS changes in living root hair cells responding to nod factors (NFs). Plant J. 56, 802–813. doi: 10.1111/j.1365-313X.2008.03644.x, PMID: 18680562

[ref14] Chabaud M. Venard C. Defaux-Petras A. Bécard G. Barker D. G. (2002). Targeted inoculation of Medicago truncatula in vitro root cultures reveals MtENOD11 expression during early stages of infection by arbuscular mycorrhizal fungi. New Phytol. 156, 265–273. doi: 10.1046/j.1469-8137.2002.00508.x 33873280

[ref15] Chen H. Richardson A. E. Rolfe B. G. (1993). Studies of the physiological and genetic basis of acid tolerance in rhizobium leguminosarum biovar trifolii. Appl. Environ. Microbiol. 59, 1798–1804. doi: 10.1128/aem.59.6.1798-1804.1993, PMID: 16348956 PMC182164

[ref16] Chen E. J. Sabio E. A. Long S. R. (2008). The periplasmic regulator ExoR inhibits ExoS/ChvI two- component signaling in *Sinorhizobium meliloti*. Mol. Microbiol. 69, 1290–1303. doi: 10.1038/jid.2014.371, PMID: 18631237 PMC2652646

[ref17] Cheng H. P. Walker G. C. (1998a). Succinoglycan is required for initiation and elongation of infection threads during nodulation of alfalfa by rhizobium meliloti. J. Bacteriol. 180, 5183–5191. doi: 10.1128/JB.180.19.5183-5191.1998, PMID: 9748453 PMC107556

[ref18] Cheng H. P. Walker G. C. (1998b). Succinoglycan production by rhizobium meliloti is regulated through the ExoS-ChvI two-component regulatory system. J. Bacteriol. 180, 20–26. doi: 10.1128/JB.180.1.20-26.1998, PMID: 9422587 PMC106843

[ref19] Cowan M. M. (1999). Plant products as antimicrobial agents. Clin. Microbiol. Rev. 12, 564–582. doi: 10.1128/CMR.12.4.564 10515903 PMC88925

[ref20] Cramer C. L. Ryder T. B. Bell J. N. Lamb C. J. (1985). Rapid switching of plant gene expression induced by fungal elicitor. Science 227, 1240–1243. doi: 10.1126/science.227.4691.1240, PMID: 17757868

[ref21] Csonka L. N. (1989). Physiological and genetic responses of bacteria to osmotic stress. Microbiol. Rev. 53, 121–147. doi: 10.1128/mr.53.1.121-147.1989 2651863 PMC372720

[ref22] Cunningham S. D. Munns D. N. (1984). The correlation between extracellular polysaccharide production and acid tolerance in rhizobium. Soil Sci. Soc. Am. J. 48, 1273–1276. doi: 10.2136/sssaj1984.03615995004800060014x

[ref23] Czernic P. Gully D. Cartieaux F. Moulin L. Guefrachi I. Patrel D.. (2015). Convergent evolution of endosymbiont differentiation in Dalbergioid and inverted repeat-lacking clade legumes mediated by nodule-specific cysteine-rich peptides. Plant Physiol. 169, 1254–1265. doi: 10.1104/pp.15.00584, PMID: 26286718 PMC4587450

[ref24] David M. Daveran M. L. Batut J. Dedieu A. Domergue O. Ghai J.. (1988). Cascade regulation of Nif gene-expression in rhizobium meliloti. Cell 54, 671–683. doi: 10.1016/S0092-8674(88)80012-6, PMID: 2842062

[ref25] Del Cerro P. Megías M. López-Baena F. J. Gil-Serrano A. Pérez-Montaño F. Ollero F. J. (2019). Osmotic stress activates nif and fix genes and induces the rhizobium tropici CIAT 899 nod factor production via NodD_2_ by up-regulation of the nodA2 operon and the nodA3 gene. PLoS One 14:e0213298. doi: 10.1371/journal.pone.0213298, PMID: 30917160 PMC6436695

[ref26] Dilworth M. J. Tiwari R. P. Reeve W. G. Glenn A. R. (2000). Legume root nodule bacteria and acid pH. Sci. Prog. 83, 357–389. PMID: 11233369

[ref27] Dixon R. Kahn D. (2004). Genetic regulation of biological nitrogen fixation. Nat. Rev. Microbiol. 2, 621–631. doi: 10.1038/nrmicro954 15263897

[ref28] Donati A. J. Jeon J. M. Sangurdekar D. So J. S. Chang W. S. (2011). Genome-wide transcriptional and physiological responses of Bradyrhizobium japonicum to paraquat-mediated oxidative stress. Appl. Environ. Microbiol. 77, 3633–3643. doi: 10.1128/AEM.00047-11, PMID: 21498770 PMC3127611

[ref29] Draghi W. O. Del Papa M. F. Hellweg C. Watt S. A. Watt T. F. Barsch A.. (2016). A consolidated analysis of the physiologic and molecular responses induced under acid stress in the legume-symbiont model-soil bacterium Sinorhizobium meliloti. Sci. Rep. 6:e29278. doi: 10.1038/srep29278, PMID: 27404346 PMC4941405

[ref30] Duc G. Trouvelot A. Gianinazzi-Pearson V. Gianinazzi S. (1989). First report of non-mycorrhizal plant mutants (Myc-) obtained in pea (Pisum sativum L.) and fababean (Vicia faba L.). Plant Sci. 60, 215–222. doi: 10.1016/0168-9452(89)90169-6

[ref31] Dylan T. Helinski D. R. Ditta G. S. (1990a). Hypoosmotic adaptation in rhizobium meliloti requires beta-(1-2)-glucan. J. Bacteriol. 172, 1400–1408. doi: 10.1128/jb.172.3.1400-1408.1990, PMID: 1689716 PMC208612

[ref32] Dylan T. Nagpal P. Helinski D. R. Ditta G. S. (1990b). Symbiotic pseudorevertants of rhizobium meliloti ndv mutants. J. Bacteriol. 172, 1409–1417. doi: 10.1128/jb.172.3.1409-1417.1990, PMID: 2307652 PMC208613

[ref33] Ehrhardt D. W. Atkinson E. M. Long S. R. (1992). Depolarization of alfalfa root hair membrane potential by rhizobium meliloti nod factors. Science 256, 998–1000. doi: 10.1126/science.10744524, PMID: 10744524

[ref34] Ehrhardt D. W. Wais R. Long S. R. (1996). Calcium spiking in plant root hairs responding to rhizobium nodulation signals. Cell 85, 673–681. doi: 10.1016/S0092-8674(00)81234-9, PMID: 8646776

[ref35] El-Yahyaoui F. Kuster H. Amor B. B. Hohnjec N. Pu A. Becker A.. (2004). Expression profiling in Medicago truncatula pdentifies more than 750 genes differentially expressed during nodulation, including many potential regulators of the symbiotic program. Plant Physiol. 136, 3159–3176. doi: 10.1104/pp.104.043612.the, PMID: 15466239 PMC523376

[ref36] Faget M. Blossfeld S. von Gillhaussen P. Schurr U. Temperton V. M. (2013). Disentangling who is who during rhizosphere acidification in root interactions: combining fluorescence with optode techniques. Front. Plant Sci. 4:392. doi: 10.3389/fpls.2013.00392, PMID: 24137168 PMC3797519

[ref37] Fedorova E. Thomson R. Whitehead L. F. Maudoux O. Udvardi M. K. Day D. A. (1999). Localization of H^+^-ATPase in soybean root nodules. Planta 209, 25–32. doi: 10.1007/s004250050603, PMID: 10467028

[ref38] Fenner B. J. Tiwari R. P. Reeve W. G. Dilworth M. J. Glenn A. R. (2004). Sinorhizobium medicae genes whose regulation involves the ActS and/or ActR signal transduction proteins. FEMS Microbiol. Lett. 236, 21–31. doi: 10.1016/j.femsle.2004.05.016 15212786

[ref39] Ferrer J. L. Austin M. B. Stewart C. Noel J. P. (2008). Structure and function of enzymes involved in the biosynthesis of phenylpropanoids. Plant Physiol. Biochem. 46, 356–370. doi: 10.1016/j.plaphy.2007.12.009, PMID: 18272377 PMC2860624

[ref40] Fonseca-García C. Nava N. Lara M. Quinto C. (2021). An NADPH oxidase regulates carbon metabolism and the cell cycle during root nodule symbiosis in common bean (Phaseolus vulgaris). BMC Plant Biol. 21, 274–290. doi: 10.1186/s12870-021-03060-z, PMID: 34130630 PMC8207584

[ref41] Fournier J. Timmers A. C. J. Sieberer B. J. Jauneau A. Chabaud M. Barker D. G. (2008). Mechanism of infection thread elongation in root hairs of *Medicago truncatula* and dynamic interplay with associated rhizobial colonization. Plant Physiol. 148, 1985–1995. doi: 10.1104/pp.108.125674, PMID: 18931145 PMC2593660

[ref42] Geddes B. A. González J. E. Oresnik I. J. (2014). Exopolysaccharide production in response to medium acidification is correlated with an increase in competition for nodule occupancy. Mol. Plant-Microbe Interact. 27, 1307–1317. doi: 10.1094/MPMI-06-14-0168-R, PMID: 25387133

[ref43] Geddes B. A. Oresnik I. J. (2016). “The mechanism of symbiotic nitrogen fixation,” in The Mechanistic Benefits of Microbial Symbionts. Advances in Environmental Microbiology. Vol. 2. Hurst (Ed.) C. J. (Switzerland: Springer International Publishing), 69–97.

[ref44] Gober J. W. Kashket E. R. (1987). K^+^ regulates bacteroid-associated functions of Bradyrhizobium. Proc. Natl. Acad. Sci. U. S. A. 84, 4650–4654. doi: 10.1073/pnas.84.13.4650, PMID: 16593858 PMC305148

[ref45] González J. E. Reuhs B. L. Walker G. C. (1996). Low molecular weight EPS II of rhizobium meliloti allows nodule invasion in Medicago sativa. Proc. Natl. Acad. Sci. U. S. A. 93, 8636–8641. doi: 10.1073/pnas.93.16.8636, PMID: 8710923 PMC38725

[ref46] Griffitts J. S. Carlyon R. E. Erickson J. H. Moulton J. L. Barnett M. J. Toman C. J.. (2008). A Sinorhizobium meliloti osmosensory two-component system required for cyclic glucan export and symbiosis. Mol. Microbiol. 69, 479–490. doi: 10.1111/j.1365-2958.2008.06304.x, PMID: 18630344

[ref47] Guefrachi I. Pierre O. Timchenko T. Alunni B. Barrière Q. Czernic P.. (2015). Bradyrhizobium BclA is a peptide transporter required for bacterial differentiation in Symbiosis with Aeschynomene legumes. Mol. Plant-Microbe Interact. 28, 1155–1166. doi: 10.1094/MPMI-04-15-0094-R, PMID: 26106901

[ref48] Guerrero-Castro J. Lozano L. Sohlenkamp C. (2018). Dissecting the acid stress response of rhizobium tropici CIAT 899. Front. Microbiol. 9:846. doi: 10.3389/fmicb.2018.00846, PMID: 29760688 PMC5936775

[ref49] Haag A. F. Baloban M. Sani M. Kerscher B. Pierre O. Farkas A.. (2011). Protection of Sinorhizobium against host cysteine-rich antimicrobial peptides is critical for symbiosis. PLoS Biol. 9:e1001169. doi: 10.1371/journal.pbio.1001169, PMID: 21990963 PMC3186793

[ref50] Harrison J. Jamet A. Muglia C. I. Sype G. Van De Aguilar O. M. Puppo A.. (2005). Glutathione plays a fundamental role in growth and symbiotic capacity of Sinorhizobium meliloti. J. Bacteriol. 187, 168–174. doi: 10.1128/JB.187.1.168, PMID: . 15601700 PMC538818

[ref51] Hartwig U. A. Maxwell C. A. Joseph C. M. Phillips D. A. (1990). Effects of alfalfa nod gene-inducing flavonoids on nodABC transcription in rhizobium meliloti strains containing different nodD genes. J. Bacteriol. 172, 2769–2773. doi: 10.1128/jb.172.5.2769-2773.1990, PMID: 2332406 PMC208924

[ref52] Hassan S. Mathesius U. (2012). The role of flavonoids in root-rhizosphere signalling: opportunities and challenges for improving plant-microbe interactions. J. Exp. Bot. 63, 3429–3444. doi: 10.1093/jxb/err430, PMID: 22213816

[ref53] Hawkins J. P. Geddes B. A. Oresnik I. J. (2017). Succinoglycan directly contributes to pH tolerance in Sinorhizboium meliloti Rm1021. Mol. Plant-Microbe Interact. 30, 1009–1019. doi: 10.1094/MPMI-07-17-0176-R, PMID: 28871850

[ref54] Heavner M. E. Qiu W. G. Cheng H. (2015). Phylogenetic co-occurrence of ExoR, ExoS, and ChvI, components of the RSI bacterial invasion switch, suggests a key adaptive mechanism regulating the transition between free-living and host-invading phases in Rhizobiales. PLoS One 10:e0135655. doi: 10.1371/journal.pone.0135655, PMID: 26309130 PMC4550343

[ref55] Heckel B. C. Tomlinson A. D. Morton E. R. Choi J. H. Fuqua C. (2014). Agrobacterium tumefaciens ExoR controls acid response genes and impacts exopolysaccharide synthesis, horizontal gene transfer, and virulence gene expression. J. Bacteriol. 196, 3221–3233. doi: 10.1128/JB.01751-14, PMID: 24982308 PMC4135700

[ref56] Hellweg C. Pühler A. Weidner S. (2009). The time course of the transcriptomic response of Sinorhizobium meliloti 1021 following a shift to acidic pH. BMC Microbiol. 9, 37–53. doi: 10.1186/1471-2180-9-37, PMID: 19216801 PMC2651895

[ref57] Hirsch A. M. Lum M. R. Downie J. A. (2001). What makes the rhizobia-legume symbiosis so special? Plant Physiol. 127, 1484–1492. doi: 10.1104/pp.010866, PMID: 11743092 PMC1540181

[ref58] Horváth B. Domonkos Á. Kereszt A. Szűcs A. Ábrahám E. Ayaydin F.. (2015). Loss of the nodule-specific cysteine rich peptide, NCR169, abolishes symbiotic nitrogen fixation in the Medicago truncatula dnf7 mutant. Proc. Natl. Acad. Sci. U. S. A. 112, 15232–15237. doi: 10.1073/pnas.1500777112, PMID: 26401023 PMC4679056

[ref59] Howieson J. G. Ewing M. A. D’Antuono M. F. (1988). Selection for acid tolerance in rhizobium meliloti. Plant Soil 105, 179–188. doi: 10.1007/BF02376781

[ref60] Hunt S. King B. J. Canvin D. T. Layzell D. B. (1987). Steady and nonsteady state gas exchange characteristics of soybean nodules in relation to the oxygen diffusion barrier. Plant Physiol. 84, 164–172. doi: 10.1104/pp.84.1.164, PMID: 16665392 PMC1056546

[ref61] Hunt S. Layzell D. B. (1993). Gas exchange of legume nodules and the regulation of nitrogenase activity. Annu. Rev. Plant Physiol. Plant Mol. Biol. 44, 483–511. doi: 10.1146/annurev.pp.44.060193.002411

[ref62] Jamet A. Mandon K. Puppo A. Hérouart D. (2007). H_2_O_2_ is required for optimal establishment of the Medicago sativa/Sinorhizobium meliloti symbiosis. J. Bacteriol. 189, 8741–8745. doi: 10.1128/JB.01130-07, PMID: 17921312 PMC2168964

[ref63] Jamet A. Sigaud S. Van de Sype G. Puppo A. Herouart D. (2003). Expression of the bacterial catalase genes during Sinorhizobium meliloti-Medicago sativa symbiosis and their crucial role during the infection process. Mol. Plant-Microbe Interact. 16, 217–225. doi: 10.1094/MPMI.2003.16.3.217, PMID: 12650453

[ref64] Jones D. L. Dennis P. G. Owen A. G. Van Hees P. A. W. (2003). Organic acid behavior in soils - misconceptions and knowledge gaps. Plant Soil 248, 31–41. doi: 10.1023/A:1022304332313

[ref65] Jones K. M. Kobayashi H. Davies B. W. Taga M. E. Walker G. C. (2007). How rhizobial symbionts invade plants: the Sinorhizobium-Medicago model. Nat. Rev. Microbiol. 5, 619–633. doi: 10.1038/nrmicro1705, PMID: 17632573 PMC2766523

[ref66] Jungk A. O. (2002). “Dynamics of nutrient movement at the soil root interface,” in Plant Roots. The Hidden Half. Kafkafi U. Waisel Y. Eshel (Eds.) A. (United States: CRC Press), 587–616.

[ref67] Kannenberg E. L. Brewin N. J. (1989). Expression of a cell surface antigen from rhizobium leguminosarum 3841 is regulated by oxygen and pH. J. Bacteriol. 171, 4543–4548. doi: 10.1128/jb.171.9.4543-4548.1989, PMID: 2768181 PMC210248

[ref68] Kawaharada Y. Kelly S. Nielsen M. W. Hjuler C. T. Gysel K. Muszyński A.. (2015). Receptor-mediated exopolysaccharide perception controls bacterial infection. Nature 523, 308–312. doi: 10.1038/nature14611, PMID: 26153863

[ref69] Keating D. H. Willits M. G. Long S. R. (2002). A Sinorhizobium meliloti lipopolysaccharide mutant altered in cell surface sulfation. J. Bacteriol. 184, 6681–6689. doi: 10.1128/JB.184.23.6681, PMID: 12426356 PMC135449

[ref70] Király Z. El-Zahaby H. M. Klement Z. (1997). Role of extracellular polysaccharide (EPS) slime of plant pathogenic bacteria in protecting cells to reactive oxygen species. J. Phytopathol. 145, 59–68. doi: 10.1111/j.1439-0434.1997.tb00365.x

[ref71] Lehman A. P. Long S. R. (2013). Exopolysaccharides from Sinorhizobium meliloti can protect against H_2_O_2_-dependent damage. J. Bacteriol. 195, 5362–5369. doi: 10.1128/JB.00681-13, PMID: 24078609 PMC3837946

[ref72] Lelpi L. Dylan T. Ditta G. S. Helinski D. R. Stanfield S. W. (1990). The ndvB locus of rhizobium meliloti encodes a 319-kDa involved in the production of β-(1-2)-glucan. J. Biol. Chem. 265, 2643–2651. 2154461

[ref73] Li L. Jia Y. Hou Q. Charles T. C. Nester E. W. Pan S. Q. (2002). A global pH sensor: agrobacterium sensor protein ChvG regulates acid-inducible genes on its two chromosomes and Ti plasmid. Proc. Natl. Acad. Sci. U. S. A. 99, 12369–12374. doi: 10.1073/pnas.192439499, PMID: 12218184 PMC129451

[ref74] Liang Y. Cao Y. Tanaka K. Thibivilliers S. Wan J. Choi J.. (2013). Nonlegumes respond to Rhizobial nod factors by suppressing the innate immune response. Science 341, 1384–1387. doi: 10.1126/science.1242736, PMID: 24009356

[ref75] Lohar D. P. Haridas S. Gantt J. S. VandenBosch K. A. (2007). A transient decrease in reactive oxygen species in roots leads to root hair deformation in the legume-rhizobia symbiosis. New Phytol. 173, 39–49. doi: 10.1111/j.1469-8137.2006.01901.x, PMID: 17176392

[ref76] Lohar D. P. Sharopova N. Endre G. Peñuela S. Samac D. Town C.. (2006). Transcript analysis of early nodulation events in Medicago truncatula. Plant Physiol. 140, 221–234. doi: 10.1104/pp.105.070326.1, PMID: 16377745 PMC1326046

[ref77] Lu H. Y. Luo L. Yang M. H. Cheng H.-P. (2012). Sinorhizobium meliloti ExoR is the target of periplasmic proteolysis. J. Bacteriol. 194, 4029–4040. doi: 10.1128/JB.00313-12, PMID: 22636773 PMC3416547

[ref78] Madsen E. B. Madsen L. H. Radutoiu S. Olbryt M. Rakwalska M. Szczyglowski K.. (2003). A receptor kinase gene of the LysM type is involved in legume perception of rhizobial signals. Nature 425, 637–640. doi: 10.1038/nature02045, PMID: 14534591

[ref79] Maillet F. Fournier J. Mendis H. C. Tadege M. Wen J. Ratet P.. (2020). Sinorhizobium meliloti succinylated high-molecular-weight succinoglycan and the Medicago truncatula LysM receptor-like kinase MtLYK10 participate independently in symbiotic infection. Plant J. 102, 311–326. doi: 10.1111/tpj.14625, PMID: 31782853 PMC9327734

[ref80] Maroti G. G. Kereszt A. Kondorosi E. Mergaert P. (2011). Natural roles of antimicrobial peptides in microbes, plants and animals. Res. Microbiol. 162, 363–374. doi: 10.1016/j.resmic.2011.02.005, PMID: 21320593

[ref81] Mendis H. C. Madzima T. F. Queiroux C. Jones K. M. (2016). Function of succinoglycan polysaccharide in Sinorhizobium meliloti host plant invasion depends on succinylation, not molecular weight. MBio 7:e00606-16. doi: 10.1128/mBio.00606-16, PMID: 27329751 PMC4916376

[ref82] Mergaert P. Nikovics K. Kelemen Z. Maunoury N. Vaubert D. Kondorosi A.. (2003). A novel family in Medicago truncatula consisting of more than 300 nodule-specific genes coding for small, secreted polypeptides with conserved cysteine motifs. Plant Physiol. 132, 161–173. doi: 10.1104/pp.102.018192, PMID: 12746522 PMC166962

[ref83] Mergaert P. Uchiumi T. Alunni B. Evanno G. Cheron A. Catrice O.. (2006). Eukaryotic control on bacterial cell cycle and differentiation in the rhizobium-legume symbiosis. Proc. Natl. Acad. Sci. U. S. A. 103, 5230–5235. doi: 10.1073/pnas.0600912103, PMID: 16547129 PMC1458823

[ref84] Miller K. J. Kennedy E. P. Reinhold V. N. (1986). Osmotic adaptation by gram-negative bacteria: possible role for periplasmic oligosaccharides. Science 231, 48–51. doi: 10.1126/science.3941890, PMID: 3941890

[ref85] Miller K. J. Wood J. M. (1996). Osmoadatptation by rhizosphere bacteria. Annu. Rev. Microbiol. 6:e23307. doi: 10.1371/journal.pone.0023307, PMID: 8905077

[ref86] Miller-Williams M. Loewen P. C. Oresnik I. J. (2006). Isolation of salt-sensitive mutants of Sinorhizobium meliloti strain Rm1021. Microbiology 152, 2049–2059. doi: 10.1099/mic.0.28937-0, PMID: 16804180

[ref87] Muglia C. I. Grasso D. H. Aguilar O. M. (2007). Rhizobium tropici response to acidity involves activation of glutathione synthesis. Microbiology 153, 1286–1296. doi: 10.1099/mic.0.2006/003483-0, PMID: 17379738

[ref88] Mylona P. Pawlowski K. Bisseling T. (1995). Symbiotic nitrogen fixation. Plant Cell 7, 869–885. doi: 10.1097/00010694-199511000-00009 12242391 PMC160880

[ref89] Nagpal P. Khanuja S. P. Stanfield S. W. (1992). Suppression of the ndv mutant phenotype of rhizobium meliloti by cloned exo genes. Mol. Microbiol. 6, 479–488. doi: 10.1111/j.1365-2958.1992.tb01492.x, PMID: 1560776

[ref90] Ojeda K. J. Simonds L. Noel K. D. (2013). Roles of predicted glycosyltransferases in the biosynthesis of the rhizobium etli CE3 O antigen. J. Bacteriol. 195, 1949–1958. doi: 10.1128/JB.02080-12, PMID: 23435981 PMC3624598

[ref91] Oldroyd G. E. D. (2013). Speak, friend, and enter: signalling systems that promote beneficial symbiotic associations in plants. Nat. Rev. Microbiol. 11, 252–263. doi: 10.1038/nrmicro2990, PMID: 23493145

[ref92] Oldroyd G. E. D. Downie J. A. (2008). Coordinating nodule morphogenesis with rhizobial infection in legumes. Annu. Rev. Plant Biol. 59, 519–546. doi: 10.1146/annurev.arplant.59.032607.092839, PMID: 18444906

[ref93] Oldroyd G. E. D. Murray J. D. Poole P. S. Downie J. A. (2011). The rules of engagement in the legume-rhizobial symbiosis. Annu. Rev. Genet. 45, 119–144. doi: 10.1146/annurev-genet-110410-132549, PMID: 21838550

[ref94] Pauly N. Pucciariello C. Mandon K. Innocenti G. Jamet A. Baudouin E.. (2006). Reactive oxygen and nitrogen species and glutathione: key players in the legume-rhizobium symbiosis. J. Exp. Bot. 57, 1769–1776. doi: 10.1093/jxb/erj184, PMID: 16698817

[ref95] Penterman J. Abo R. P. De Nisco N. J. Arnold M. F. F. Longhi R. Zanda M.. (2014). Host plant peptides elicit a transcriptional response to control the Sinorhizobium meliloti cell cycle during symbiosis. Proc. Natl. Acad. Sci. U. S. A. 111, 3561–3566. doi: 10.1073/pnas.1400450111, PMID: 24501120 PMC3948309

[ref96] Pérez-Montaño F. del Cerro P. Jiménez-Guerrero I. López-Baena F. J. Cubo M. T. Hungria M.. (2016). RNA-seq analysis of the rhizobium tropici CIAT 899 transcriptome shows similarities in the activation patterns of symbiotic genes in the presence of apigenin and salt. BMC Genomics 17, 198–111. doi: 10.1186/s12864-016-2543-3, PMID: 26951045 PMC4782375

[ref97] Pierre O. Engler G. Hopkins J. Brau F. Boncompagni E. Hérouart D. (2013). Peribacteroid space acidification: A marker of mature bacteroid functioning in Medicago truncatula nodules. Plant Cell Environ. 36, 2059–2070. doi: 10.1111/pce.12116, PMID: 23586685

[ref98] Pueppke S. G. Broughton W. J. (1999). Rhizobium sp. strain NGR234 and R. fredii USDA257 share exceptionally broad, nested host ranges. Mol. Plant-Microbe Interact. 12, 293–318. doi: 10.1094/MPMI.1999.12.4.293, PMID: 10188270

[ref99] Puppo A. Pauly N. Boscari A. Mandon K. Brouquisse R. (2013). Hydrogen peroxide and nitric oxide: key regulators of the legume-rhizobium and mycorrhizal symbioses. Antioxid. Redox Signal. 18, 2202–2219. doi: 10.1089/ars.2012.5136, PMID: 23249379

[ref100] Pusztahelyi T. (2018). Chitin and chitin-related compounds in plant–fungal interactions. Mycology 9, 189–201. doi: 10.1080/21501203.2018.1473299, PMID: 30181925 PMC6115883

[ref101] Ramu S. K. Peng H. M. Cook D. R. (2002). Nod factor induction of reactive oxygen species production is correlated with expression of the early nodulin gene rip1 in Medicago truncatula. Mol. Plant-Microbe Interact. 15, 522–528. doi: 10.1094/MPMI.2002.15.6.522, PMID: 12059100

[ref102] Reeve W. G. Dilworth M. J. Tiwari R. P. Glenn A. R. (1997). Regulation of exopolysaccharide production in rhizobium leguminosarum biovar viciae WSM710 involves exoR. Microbiology 143, 1951–1958. doi: 10.1099/00221287-143-6-1951, PMID: 9202471

[ref103] Reuber T. L. Urzainqui A. Glazebrook J. Reed J. W. Walker G. C. (1990). Genetic analyses and manipulations of rhizobium meliloti exopolysaccharides. Nov. Biodegrad. Microb. Polym. 12, 285–294. doi: 10.1007/978-94-009-2129-0_24

[ref104] Reyes-González A. Talbi C. Rodríguez S. Rivera P. Zamorano-Sánchez D. Girard L. (2016). Expanding the regulatory network that controls nitrogen fixation in Sinorhizobium meliloti: elucidating the role of the two-component system hFixL-FxkR. Microbiology 162, 979–988. doi: 10.1099/mic.0.000284, PMID: 27010660

[ref105] Riccillo P. M. Muglia C. I. De Bruijn F. J. Roe A. J. Booth I. R. Aguilar O. M. (2000). Glutathione is involved in environmental stress responses in rhizobium tropici, including acid tolerance. J. Bacteriol. 182, 1748–1753. doi: 10.1128/JB.182.6.1748-1753.2000, PMID: 10692382 PMC94474

[ref106] Rutten P. J. Steel H. Hood G. A. Ramachandran V. K. McMurtry L. Geddes B.. (2021). Multiple sensors provide spatiotemporal oxygen regulation of gene expression in a rhizobium-legume symbiosis. PLoS Genet. 17:e1009099. doi: 10.1371/journal.pgen.1009099, PMID: 33539353 PMC7888657

[ref107] Santos R. Hérouart D. Sigaud S. Touati D. Puppo A. (2001). Oxidative burst in alfalfa- Sinorhizobium meliloti symbiotic interaction. Mol. Plant-Microbe Interact. 14, 86–89. doi: 10.1094/MPMI.2001.14.1.86, PMID: 11194876

[ref108] Schopfer C. R. (1999). The male determinant of self-incompatibility in brassica. Science 286, 1697–1700. doi: 10.1126/science.286.5445.1697, PMID: 10576728

[ref109] Shaw S. L. Long S. R. (2003). Nod factor inhibition of reactive oxygen efflux in a host legume. Plant Physiol. 132, 2196–2204. doi: 10.1104/pp.103.021113, PMID: 12913174 PMC181303

[ref110] Smith L. T. Allaith A. A. Smith G. M. (1994). Mechanism of osmotically regulated N-acetylglutaminylglutamine amide production in rhizobium meliloti. Plant Soil 161, 103–108. doi: 10.1007/BF02183090

[ref111] Streng A. Op Den Camp R. Bisseling T. Geurts R. Camp R. Den Bisseling T.. (2011). Evolutionary origin of rhizobium nod factor signaling. Plant Signal. Behav. 6, 1510–1514. doi: 10.4161/psb.6.10.17444, PMID: . 21904113 PMC3256379

[ref112] Tang Y. Hollingsworth R. I. (1998). Regulation of lipid synthesis in Bradyrhizobium japonicum: low oxygen concentrations trigger phosphatidylinositol biosynthesis. Appl. Environ. Microbiol. 64, 1963–1966. doi: 10.1128/AEM.64.5.1963-1966.1998, PMID: 9572982 PMC106261

[ref113] Tóth K. Stacey G. (2015). Does plant immunity play a critical role during initiation of the legume-rhizobium symbiosis? Front. Plant Sci. 6, 401–408. doi: 10.3389/fpls.2015.00401, PMID: 26082790 PMC4451252

[ref114] Van de Velde W. Zehirov G. Szatmari A. Debreczeny M. Ishihara H. Kevei Z.. (2010). Plant peptides govern terminal differentiation of bacteria in symbiosis. Science 327, 1122–1126. doi: 10.1126/science.1184057, PMID: 20185722

[ref115] Via V. D. Zanetti M. E. Blanco F. (2016). How legumes recognize rhizobia. Plant Signal. Behav. 11:e1120396. doi: 10.1080/15592324.2015.1120396, PMID: 26636731 PMC4883929

[ref116] Virts E. L. Stanfield S. W. Helinski D. R. Ditta G. S. (1988). Common regulatory elements control symbiotic and microaerobic induction of nifA in rhizobium meliloti. Proc. Natl. Acad. Sci. U. S. A. 85, 3062–3065. doi: 10.1073/pnas.85.9.3062, PMID: 2834732 PMC280143

[ref117] Winkel-Shirley B. (2001). Flavonoid biosynthesis. A colorful model for genetics, biochemistry, cell biology, and biotechnology. Plant Physiol. 126, 485–493. doi: 10.1104/pp.126.2.485, PMID: 11402179 PMC1540115

[ref118] Yancey P. H. Clark M. E. Hand S. C. Bowlus R. D. Somero G. N. (1982). Living with water stress: evolution of osmolyte systems. Science 217, 1214–1222. doi: 10.1126/science.7112124, PMID: 7112124

[ref119] Yao S. Y. Luo L. Har K. J. Becker A. Rüberg S. Yu G. Q.. (2004). Sinorhizobium meliloti ExoR and ExoS proteins regulate both succinoglycan and flagellum production. J. Bacteriol. 186, 6042–6049. doi: 10.1128/JB.186.18.6042-6049.2004, PMID: 15342573 PMC515170

[ref120] York W. S. McNeil M. Darvill A. G. Albersheim P. (1980). Beta-2-linked glucans secreted by fast-growing species of rhizobium. J. Bacteriol. 142, 243–248. doi: 10.1128/jb.142.1.243-248.1980, PMID: 7372570 PMC293938

[ref121] York G. M. Walker G. C. (1997). The rhizobium meliloti exoK gene and prsD/prsE/exsH genes are components of independent degradative pathways which contribute to production of low-molecular-weight succinoglycan. Mol. Microbiol. 25, 117–134. doi: 10.1046/j.1365-2958.1997.4481804.x, PMID: 11902715

[ref122] Zamorano-Sánchez D. Girard L. (2015). “FNR-like proteins in rhizobia: past and future,” in Biology Nitrogen Fixation. Brujin (Ed.) F. J. (United States: John Wiley & Sons), 155–166.

[ref123] Zhou P. Silverstein K. A. Gao L. Walton J. D. Nallu S. Guhlin J.. (2013). Detecting small plant peptides using SPADA (small peptide alignment discovery application). BMC Bioinfo. 14, 335–351. doi: 10.1186/1471-2105-14-335, PMID: 24256031 PMC3924332

[ref124] Zipfel C. Oldroyd G. E. D. (2017). Plant signalling in symbiosis and immunity. Nature 543, 328–336. doi: 10.1038/nature22009, PMID: 28300100

